# Ferroptosis-related gene ATG5 is a novel prognostic biomarker in nasopharyngeal carcinoma and head and neck squamous cell carcinoma

**DOI:** 10.3389/fbioe.2022.1006535

**Published:** 2022-09-15

**Authors:** Ming Shi, Jiangnan Du, Jingjing Shi, Yunchuanxiang Huang, Yan Zhao, Lan Ma

**Affiliations:** ^1^ Institute of Biopharmaceutical and Health Engineering, Tsinghua Shenzhen International Graduate School, Tsinghua University, Shenzhen, China; ^2^ Dongguan Key Laboratory of Medical Bioactive Molecular Developmental and Translational Research, Guangdong Provincial Key Laboratory of Medical Molecular Diagnostics, Guangdong Medical University, Dongguan, China; ^3^ Tsinghua-Berkeley Shenzhen Institute, Tsinghua University, Shenzhen, China; ^4^ Shenzhen Bay Laboratory, Institute of Biomedical Health Technology and Engineering, Shenzhen, China

**Keywords:** ferroptosis-related genes, immune infiltration, nasopharyngeal carcinoma, head and neck squamous cell carcinoma, the Cancer Genome Atlas

## Abstract

Nasopharyngeal carcinoma (NPC), a subtype of head and neck squamous cell carcinoma (HNSCC), is a malignant tumor that originates in the mucosal epithelium of the nasopharynx. Ferroptosis plays a key role in tumor suppression, while its prognostic value and critical factors in NPC have not been further explored. We select the Cancer Genome Atlas (TCGA) HNSCC dataset and the Gene Expression Omnibus (GEO) dataset of NPC samples, and find that ferroptosis-related factor ATG5 shows a high expression level with poor overall survival (OS) in HNSCC and NPC samples and is positively correlated with PD-L1/PD-L2 expression (*p* < 0.05). Furthermore, ATG5 high expression HNSCC patients show poor efficacy and short survival after receiving immune checkpoint blockade therapy treatment (*p* < 0.05). Moreover, ATG5 is significantly positively correlated with G2M checkpoint pathway (*ρ*_Spearman_ = 0.41, *p* < 0.01), and G2M checkpoint inhibitor drugs have lower IC_50_ in HNSCC patients with high expression of ATG5 (*p* < 0.01), indicating the potential value of G2M inhibitors in HNSCC/NPC treatment. In summary, our study shows that ferroptosis-related factors play a key role in immune infiltration in NPC and HNSCC, and ATG5, as a key immune invasion-related ferroptosis-related factor, has the potential to be a novel prognostic biomarker and a potential target in therapy for NPC and HNSCC.

## Introduction

Nasopharyngeal carcinoma (NPC) is a type of head and neck squamous cell carcinoma (HNSCC) originating in the epithelial tissue of the nasopharynx (Lo and Huang 2002). Within addition, it is frequently an Epstein-Barr virus (EBV)-associated epithelial malignancy. Unlike other HNSCCs, the occurrence of NPC has a distinct ethnic and regional predisposition, mainly endemic in southern China, southeast Asia, north Africa, and the Arctic (Ma, Hui et al., 2017). NPC causes 50,000 deaths each year (Mahdavifar, Ghoncheh et al., 2016; Shivappa, Hebert et al., 2016), with the majority of new and fatal cases concentrated in China (Torre, Bray et al., 2015; Chen, Zheng et al., 2016). Early treatment efficacy rates for NPC are high, with a 10-year survival rate of more than 90% for stage I and more than 50% for stage II (Chua, Sham et al., 2003). However, most patients are already in advanced stage of NPCs when diagnosis, accounting for 60%–70% of total NPCs (Chen, L et al., 2012). At the same time, 10%–40% of patients with cured NPC show recurrence (Yu, Xia et al., 2016). Due to the late presentation of lesions, accompanied by some degree of cancer cell metastasis, the response of available therapies is poor, and the median survival in advanced patients is only 3 years (Al-Sarraf, LeBlanc et al., 1998). Studies have found that NPC was characterized highly infiltrated immune cells around and inside tumor lesions (Wang, Chen et al., 2018; Chen, Yin et al., 2020). Although immunotherapy has been introduced as treatment for NPC, there is still a need to improve the therapeutic effect (Hsu, Lee et al., 2017; Ma, Lim et al., 2018).

Ferroptosis is a cell death pathway that relies on iron ions to product reactive oxygen species (ROS), which causes the accumulation of lipid peroxide, and eventually damages the cell membrane structure, resulting in cell death (Stockwell, Friedmann Angeli et al., 2017). Cancer cells require and contain more iron ions than normal cells (Liang, Zhang et al., 2019). Through this differentiated property, iron ions can be induced to promote ferroptosis in cancer cells, achieving precise treatment of cancer (Hassannia, Vandenabeele et al., 2019). It was found that NPC cells could be killed by the induction of ferroptosis pathways, and they were most sensitive to ferroptosis inducers (Huang, Cao et al., 2021). According to the report, during cancer immunotherapy, ferroptosis can be activated by interferon gamma (IFNγ) released from CD8^+^ T cells, and IFNγ down-regulates the expression of SLC3A2 and SLC7A11 to reduce the uptake of cystine in tumor cells, leading to ferroptosis in tumor cells (Wang, Green et al., 2019). Thus, ferroptosis may be a significant factor of NPC immunotherapy. However, the role of ferroptosis in NPC immunotherapy still needs more comprehensive studies.

In order to better understand the mechanisms of ferroptosis in NPC immunotherapy and explore more efficiency treatment for NPC, in this study, we selected the clinical data of HNSCC in the Cancer Genome Atlas (TCGA) to screen out the differential ferroptosis-related genes, and verified them by generating the expression data of NPC samples in the Gene Expression Omnibus (GEO). At the same time, aiming at the high association between NPC and immune microenvironment, we further studied NPC immune microenvironment through ferroptosis-related genes. Our data and studies have shown that the ferroptosis-related genes possessed high value in targeted drug development in NPC.

## Materials and methods

### Data acquisition

RNA-sequencing expression (level 3) profiles and corresponding clinical information for HNSCC were downloaded from TCGA dataset (https://portal.gdc.com). GSE12452 and GSE53819 microarray datasets were downloaded from the GEO database (https://www.ncbi.nlm.nih.gov/geo/). The extracted data were normalized by log2 transformation. The GEO microarray data were normalized by the normalize quantiles function of the “preprocessCore” package in R software (v3.4.1).

### Bioinformatic analysis

The “limma” package in the R software (v4.0.3) was used to study the differentially expressed mRNA. “Adjusted *p* < 0.05 and Log2 (Fold Change) > 1 or Log2 (Fold Change) < −1” were defined as the threshold for the differential expression of mRNAs. Consistency analysis using “ConsensusClusterPlus” R package (v1.54.0) was performed; the maximum number of clusters was 6, 80% of the total sample was drawn 100 times, and “pheatmap” (v1.0.12) package was used for clustering heatmaps. The “immuneeconv” package was used to assess the results of immune score evaluation. Potential immune checkpoint blockade (ICB) response was predicted with the Tumor Immune Dysfunction and Exclusion (TIDE) algorithm using “ggplot2” (v3.3.3) and “ggpubr” (0.4.0) packages in the R software (v4.0.3). To evaluate the correlations between individual genes and pathway scores, “GSVA” package was used; Parameter was chosen as method = ‘ssgsea’ and scores were analyzed by Spearman correlation. The chemotherapeutic response for each sample was predicted based on the Genomics of Drug Sensitivity in Cancer (GDSC) (https://www.cancerrxgene.org/), used by “pRRophetic” package. All parameters were set as the default values. Using the batch effect of combat and tissue type of all tissues, the duplicate gene expression was summarized as a mean value.

### Statistical analysis

Kaplan-Meier survival analysis of the different groups of samples from TCGA datasets was performed. Comparison among different groups was performed by log-rank test. The median survival time (LT50) for different groups was determined. For Kaplan-Meier curves, *p*-values and hazard ratio (HR) with 95% confidence interval (CI) were generated by log-rank tests and univariate Cox proportional hazards regression. The samples’ half-maximal inhibitory concentration (IC_50_) was estimated by ridge regression. All parameters were set as the default values. Using the batch effect of combat and tissue type of all tissues, the duplicate gene expression was summarized as a mean value. The statistical difference between two groups was compared through the Wilcox test, and significance differences between the three groups were assessed using the Kruskal–Wallis test.

## Results

### Differences in the expression of ferroptosis-related genes in NPC-affiliated HNSCC and normal tissues

**FIGURE 1:**
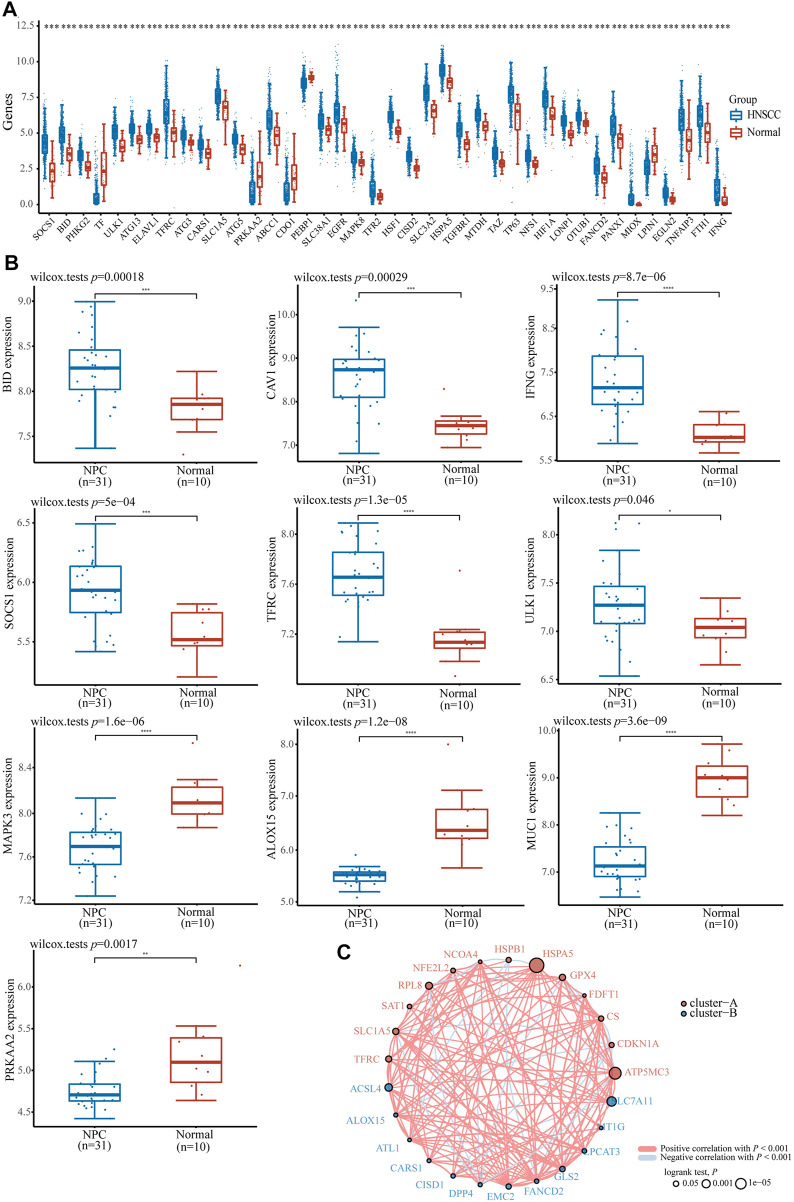
The expression distribution of ferroptosis-related gene in HNSCC and NPC patients. **(A)** ferroptosis-related gene in HNSCC and normal samples base on TCGA HNSCC dataset. **(B)** The expression distribution of ferroptosis-related gene in NPC and normal samples base on GSE12452 dataset. **(C)** The correlation of ferroptosis-related gene in HNSCC samples, circles represent the ferroptosis-related gene, red and blue represent positive and negative correlation between each gene, the size of the circle represent log rank *p*. *p < 0.05, **p < 0.01, ***p < 0.001.

To understand the role of ferroptosis-related genes in NPC, we investigated differences in the expression of ferroptosis-related genes between cancer tissues and normal tissues using TCGA HNSCC dataset and the GEO dataset of NPC samples. First, we downloaded and analyzed the expression profiles of 504 HNSCC and 44 normal cases and found that 69 genes, including *GPX4*, *BID*, *PHKG2*, *ULK1*, *ATG13*, *TFRC*, *ATG3*, *CARS1*, *SLC1A5*, *SOCS1*, *MAPK8*, *TFR2*, and others, were significantly highly expressed (*p* < 0.05); *TF*, *PRKAA2*, *CDO1*, *PEBP1*, *LPIN1*, and other 15 genes were significantly expressed at low levels (*p* < 0.05, Figure 1A); the expression of other ferroptosis-related genes were not significantly different between the tissues (*p* > 0.05, Supplementary Table S1); we found a similar trend in the GSE12452 dataset (Figure 1B). At the same time, most ferroptosis-related genes were positively correlated with HNSCC in prognosis analysis (Figure 1C). Taken together, these findings suggest that ferroptosis-related genes may play a key physiological role in the development of NPC and HNSCC.

### Relationship between ferroptosis-related genes and immune cell infiltration in NPC-affiliated HNSCC

**FIGURE 2:**
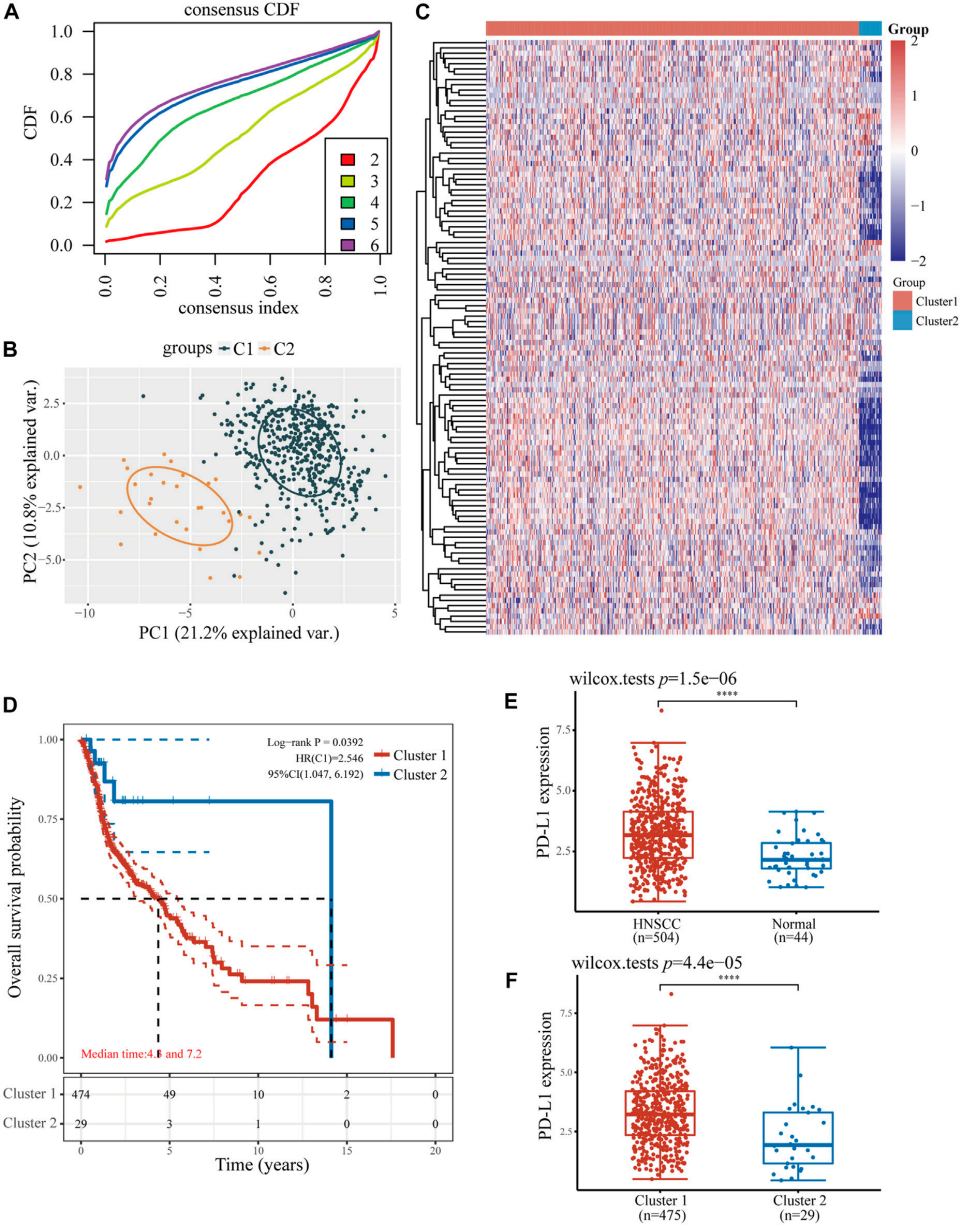
The relationship between ferroptosis-related genes and immune cell infiltration in two HNSCC subtypes. **(A)** The consensus clustering cumulative distribution function curves for *k* = 2–6. **(B)** The PCA of two HNSCC clusters. **(C)** The consistency of clustering results heatmap (*k* = 2), rows and columns represent samples, the different colors represent different types. **(D)** The Kaplan–Meier survival analysis of two clusters of samples from TCGA HNSCC dataset. **(E)** The expression level of PD-L1 in HNSCC and normal samples. **(F)** The expression level of PD-L1 in two clusters of HNSCC samples. *p < 0.05, **p < 0.01, ***p < 0.001.

**FIGURE 3:**
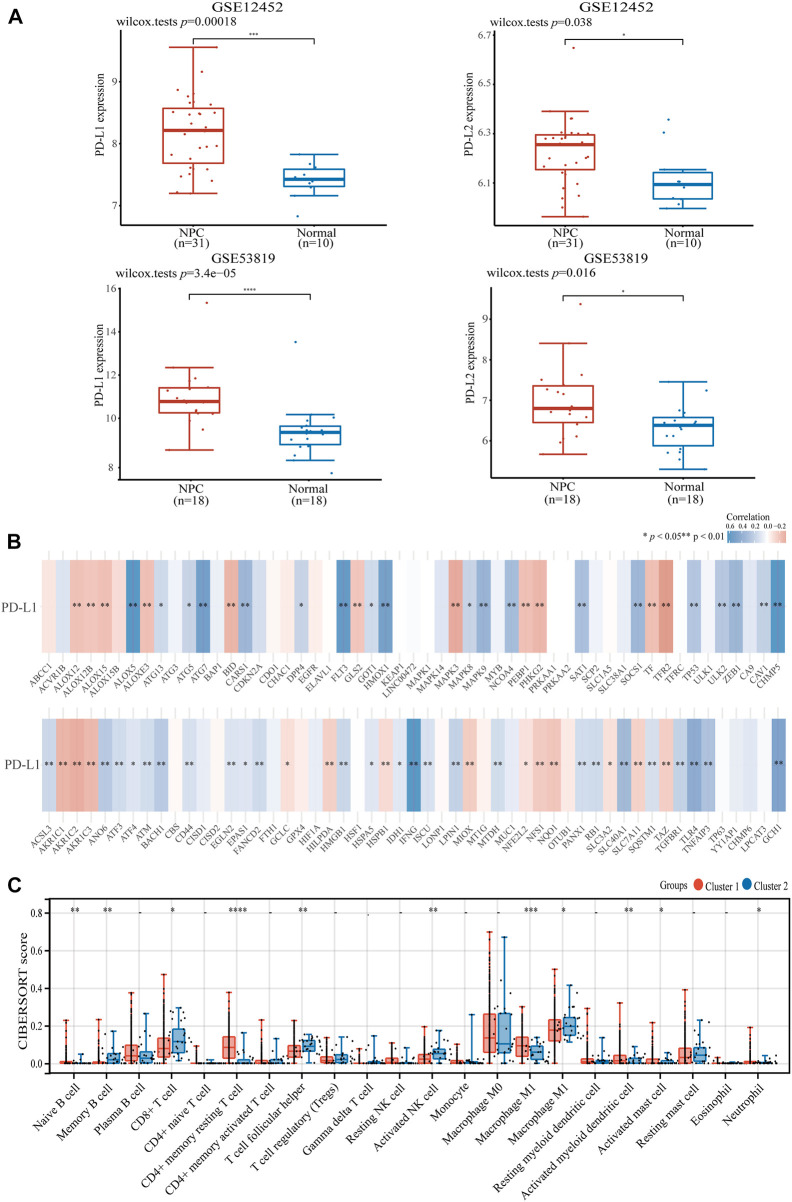
The expression of PD-L1 and PD-L2 in HNSCC and NPC patients. **(A)** The expression of PD-L1/PD-L2 in NPC and normal samples base on GSE12452 and GSE53819 dataset. **(B)** The correlation of PD-L1 with ferroptosis-related genes in TCGA HNSCC dataset. **(C)** The expression distribution of CIBERSORT immune score in two clusters of HNSCC samples. *p < 0.05, **p < 0.01, ***p < 0.001.

Considering NPC is highly infiltrated by immune cells and ferroptosis was activated by immunotherapy, we studied the relationship between ferroptosis-related genes and immune cell infiltration. Based on the expression level of ferroptosis-related genes, we first divided 504 patients with HNSCC into two groups, one with 475 patients and the other with 29 patients (Figure 2A) by R packet, and analyzed the gene expression profiles between the two groups (Figure 2B) by principal component analysis (PCA) method. The results showed that there were differences in gene expression between the two groups. Most ferroptosis-related genes were expressed at low levels in group 2 (Figure 2C), which had a better overall survival (OS) (*p* < 0.05, Figure 2D). Then we studied the expression of PD-L1 and PD-L2 in patients with HNSCC. It was shown that both PD-L1 and PD-L2 were overexpressed in HNSCC tissues and group 1 patients (*p* < 0.01, Figures 2E,F; Supplementary Figures S1A,B). Similar trends were found in two GSE datasets of NPCs (PD-L1, *p* < 0.01; PD-L2, *p* < 0.05, Figure 3A). Further analysis found that the expression of PD-L1 was positively correlated with the expression of 44 ferroptosis-related genes and negatively correlated with that of 24 genes in HNSCC tissues (*p* < 0.05, Figure 3B). Interestingly, PD-L2 expression was also positively correlated with that of 44 ferroptosis-related genes, although the specific genes are not same as PD-L1, 29 genes were negatively correlated with that of PD-L2 (*p* < 0.05, Supplementary Figure S1C). In the relationship between ferroptosis-related genes and HNSCC immune microenvironment, we found different ferroptosis-related gene expression level affect different immune cell types (Figure 3C). Naïve B cell, memory B cell, memory resting CD4^+^ T cell, resting NK cell, gamma delta T cell, and macrophage M1 have higher activity (*p* < 0.05) in group 1 patients, while CD8^+^ T cell, T cell follicular helper, and activated NK cell have high activity in group 2 patients, respectively (*p* < 0.05).

### The ferroptosis-related gene ATG5 is overexpressed in NPC and can be used as a significant independent prognostic marker

**FIGURE 4:**
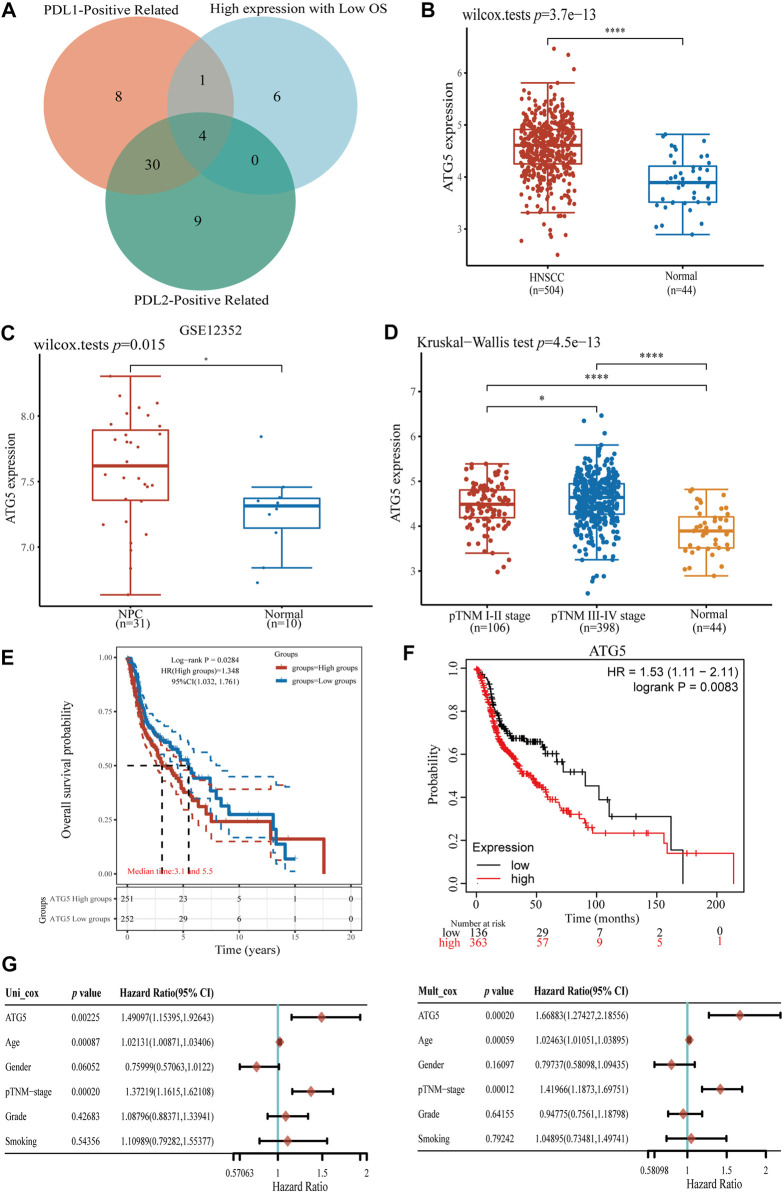
Analysis of ferroptosis-related gene ATG5 expression in HNSCC and NPC. **(A)** The Venn diagram of PD-L1/PD-L2 positive ferroptosis-related gene and high expression with OS in HNSCC samples, four ferroptosis-related genes were conformed. **(B)** ATG5 gene up-expression in HNSCC samples compared with normal samples. **(C)** ATG5 gene also highly expressed in NPC GSE12352 dataset **(D)** Boxplots of expression of ATG5 gene in different stage of HNSCC. **(E,F)** The Kaplan–Meier analysis of high and low ATG5 expression level in TCGA HNSCC dataset. **(G)** Forest plots of univariate and multivariate Cox regression of ATG5 expression and other clinicopathological factors. *p < 0.05, **p < 0.01, ***p < 0.001.

To further explore the role of ferroptosis-related genes in NPC development and tumor immune infiltration, we screened ferroptosis-related genes with high-expression, low OS, and positive association with PD-L1/PD-L2 expression in HNSCC. A comparison of the two groups showed that ATG5 is the key ferroptosis-related gene, which was not only highly expressed and had worse OS in HNSCC, but also positively correlated with PD-L1 and PD-L2 expression (*p* < 0.05, Figure 4A). In addition, ATG5 was significantly highly expressed in the GSE dataset for NPC (*p* < 0.05, Figure 4B). We also observed high ATG5 expression in late-stage HNSCC patients, with a *p* < 0.05 (Figure 4C). High expression of ATG5 was found in HNSCC patients with a worse OS (*p* < 0.05, Figure 4D), similar result also can be found in the Kaplan-Meier analysis (*p* < 0.05, Figure 4E). Using Cox analysis, we found in univariate and multivariate analysis, age, TNM staging, and ATG5 expression were significantly correlated with HNSCC OS (*p* < 0.01, Figure 4F). Those results suggested that ferroptosis-related ATG5 plays an important role in tumor development and immune infiltration in NPC and HNSCC, and thus, ATG5 could be used as an independent prognostic marker for NPC and HNSCC patients.

### Relationship of ATG5 expression with ICB and immune cell infiltration

**FIGURE 5:**
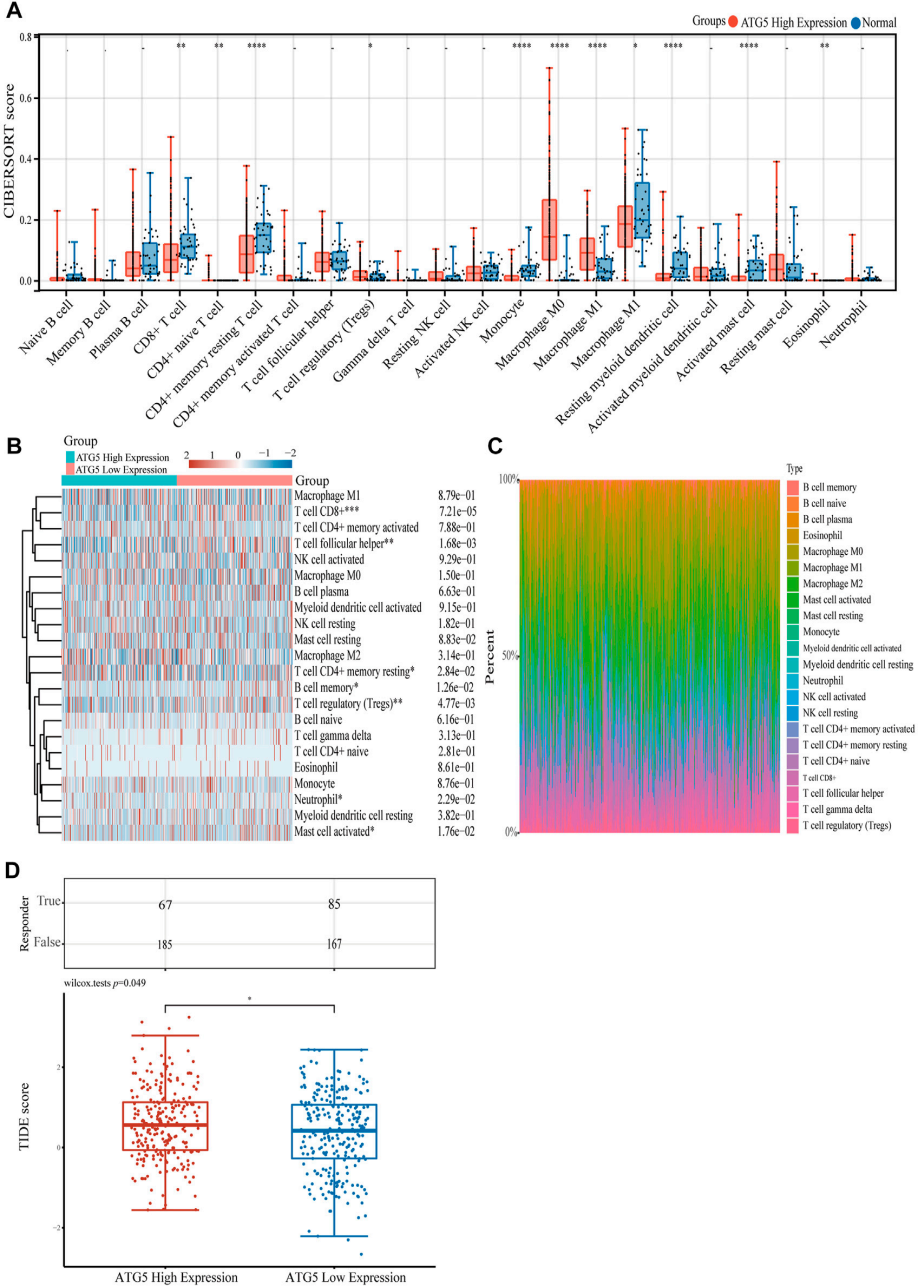
The relationship of ATG5 expression with immune cell infiltration. **(A)** The expression distribution of CIBERSORT immune score in ATG5 high expression HNSCC samples and normal samples. **(B)** Immune cell score heatmap of ATG5 high expression and ATG5 low expression in HNSCC samples. **(C)** The percentage abundance of tumor infiltrating immune cells between ATG5 high expression and ATG5 low expression HNSCC samples. **(D)** The distribution of immune response scores in ATG5 high expression and ATG5 low expression HNSCC samples in the prediction results. *p < 0.05, **p < 0.01, ***p < 0.001.

To gain a better understanding of the function of ATG5 in NPC immune cell infiltration, further studies showed that in HNSCC samples with high expression of ATG5, CD8^+^ T cell, memory resting CD4^+^ T cell, activated mast cell, monocyte, macrophage M2, and other immune cells were significantly reduced, and only T cell regulatory (Tregs) and macrophage M0 cells increased significantly, respectively (*p* < 0.05, Figure 5A). A heatmap showed trends and percentages of tumor-infiltrating immune cells in high and low ATG5 expression HNSCC samples (Figures 5B,C). To further understand the effect of ATG5 in HNSCC, based on expression profiling data, we used the TIDE algorithm to predict the responsiveness of high and low ATG5 expression groups in HNSCC to immune checkpoint inhibitors. The results showed that ATG5 high expression HNSCC patients had a higher TIDE score (*p* < 0.05, Figure 5D), predicted poor efficacy of ICB therapy, and poor survival after receiving ICB treatment.

### ATG5 is significantly associated with the G2M checkpoint pathway in HNSCC

**FIGURE 6:**
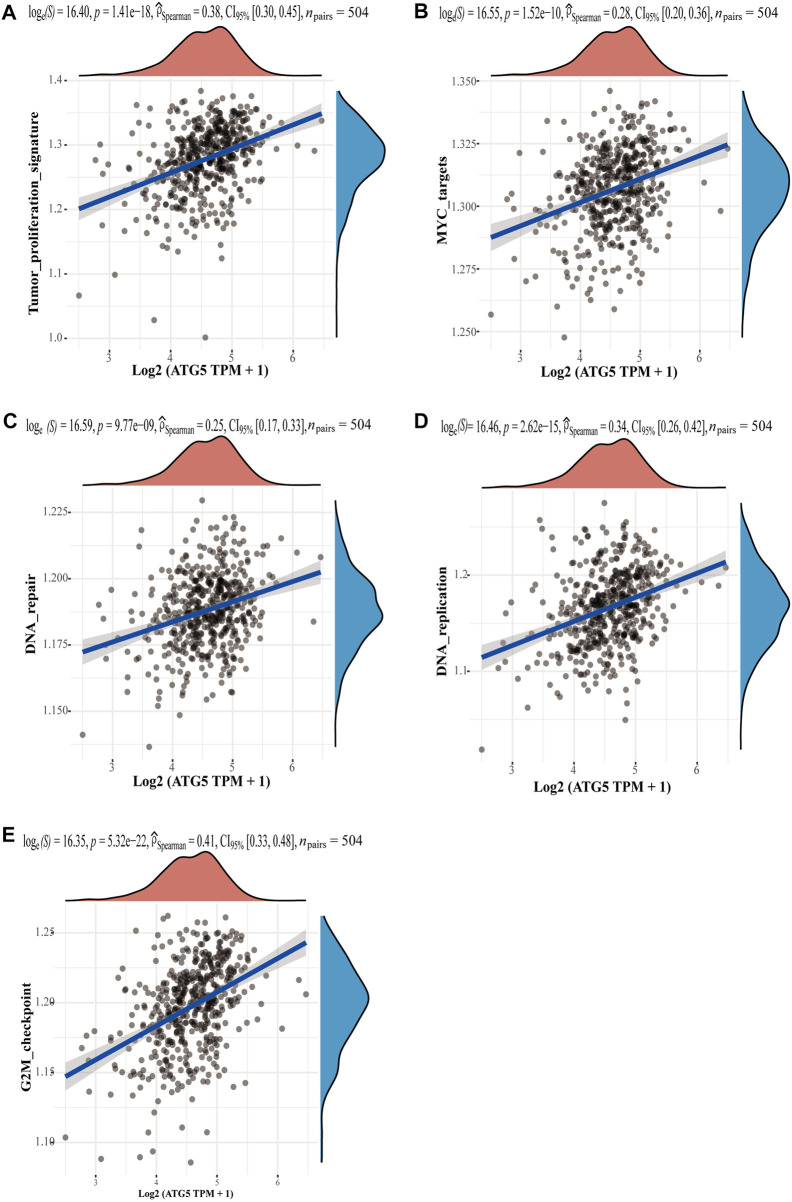
The correlations between ATG5 and pathways. **(A)** Tumor proliferation signatures, **(B)** MYC targets, **(C)** DNA repair, **(D)** DNA replication, and **(E)** G2M checkpoint. The abscissa represents the distribution of the gene expression, and the ordinate represents the distribution of the pathway score.

**FIGURE 7:**
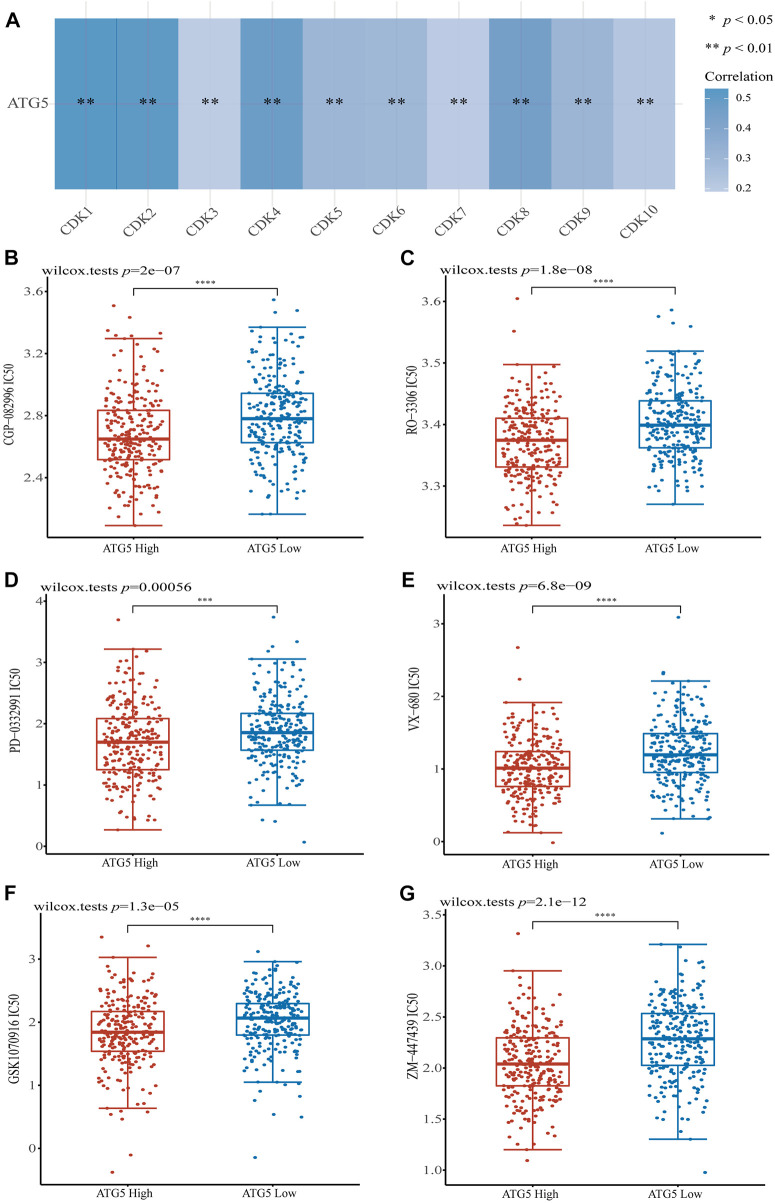
The correlations between ATG5 and G2M checkpoint. **(A)** The correlation of ATG5 and CDK family genes in TCGA HNSCC dataset. G2M checkpoint inhibitors **(B)** CGP-082996, **(C)** RO-3306, **(D)** PD-0332991, **(E)** VX-680, **(F)** GSK1070916 and **(G)** ZM-447439 had lower IC_50_ in ATG5 high expression HNSCC samples. *p < 0.05, **p < 0.01, ***p < 0.001.

Given the important role of the ferroptosis-related gene ATG5 in NPC and HNSCC, we obtained the relationship between ATG5 and different pathways by single sample gene set enrichment analysis (ssGSEA) method, which calculated the correlation score between ATG5 gene and pathways in each HNSCC samples. The results demonstrated that ATG5 expression was significantly positively correlated with tumor proliferation signatures, MYC targets, DNA repair, DNA replication, and G2M checkpoint in HNSCC (*p* < 0.05, Figures 6A–E), and the Spearman coefficients were 0.38, 0.28, 0.25, 0.34, and 0.41, respectively. The CDK family genes are important regulators of G2M checkpoint, and ATG5 expression was also positively correlated with CDK1-10 expression in HNSCC (*p* < 0.01, Figure 7A). Based on this result, we performed drug therapy response prediction of high and low ATG5 expression in transcriptome of HNSCC samples, and found that the IC50 of G2M checkpoint inhibitors were lower in ATG5 high expression HNSCC samples (*p* < 0.05, Figures 7B–G), which showed a high sensitivity to G2M checkpoint inhibitors.

## Discussion

Head and neck cancer refers to tumors and tumor-like conditions in anatomical areas of the head and neck. These anatomical areas include the oral cavity, paranasal sinuses, nasopharynx, larynx, pharynx, thyroid gland and its associated lymph nodes among others. Most head and neck cancers originate from the mucosal epithelium of the mouth, pharynx, and larynx and are collectively known as HNSCC (Johnson, Burtness et al., 2020), clinic information for HNSCC patients showed in Supplementary Table S2. NPC is the most aggressive epithelial tumor in HNSCC, with a high rate of aggressiveness and high incidence of metastasis (Cramer, Burtness et al., 2019; Jicman Stan et al., 2022). The incidence and development of NPC has a distinct regional pattern, with more than 70% of new cases concentrated in East and South-East Asia (Chen, Chan et al., 2019); researches on NPC in these regions are far less compared to other global high-prevalence cancers. Here, we used NPC-affiliated HNSCC database to study the role of ferroptosis-related genes in HNSCC and NPC, and found that it is possible ferroptosis-related genes affect the tumor immune microenvironment of NPC. Through screening, we found the ferroptosis-related gene ATG5 could serve as a significant independent prognostic marker. ATG5 is not only overexpressed with low OS, but also positive correlated with PD-L1/PD-L2 expression in HNSCC and NPC. Furthermore, ATG5 has a strong correlation with multiple pathways in HNSCC and affects the therapeutic efficacy of ICB therapy. In addition, the drug IC_50_ prediction showed ATG5 high expression HNSCC samples were more sensitive to G2M checkpoint inhibitors.

Ferroptosis is an iron-dependent regulatory process of cell death caused by excessive lipid peroxidation, and the occurrence and therapeutic effect of different types of tumors are related to ferroptosis (Chen, Kang et al., 2021). In this study, HNSCC patients were divided into two groups based on the characteristics of ferroptosis-related genes. Group two had a lower OS and higher correlation with CD8^+^ T cells and activated NK cells by CIBERSORT analysis which is a method to characterize complex tissue cell composition from gene expression profiles (Newman, Liu et al., 2015). In addition, the low-expression of PD-L1/PD-L2 in group two was beneficial to recover T cell tumor response. The abundance of CD8^+^ T cell is generally closely related to the prognosis of variety of tumor patients. CD8^+^ T cells kill target cells by secreting perforin, or indirectly releasing cytokines such as tumor necrosis factor alpha (TNFα) (Zhang and Bevan 2011; Deng, Luo et al., 2019). The study found that CD8^+^ T cells screen and eliminate invaders, and if CD8^+^ T cell infiltration is less than 2.2% in tumor, the risk of developing disease progression after surgery will be 4-fold higher (Jansen, Prokhnevska et al., 2019). Activated natural killer (NK) cells also produce cytokines such as TNFα and IFNγ and inhibit cancer angiogenesis and proliferation (Whiteside 2020; Wang, Chen et al., 2021).

The better OS and immune infiltration levels in group 2 suggested the important role of ferroptosis-related genes in HNSCC and NPC, therefore, we further screened poor prognosis of ferroptosis-related genes. Finally, ATG5 was found not only to be significantly highly expressed in HNSCC and NPC, but also showed a low prognosis and positively correlation with PD-L1/PD-L2 expression. ATG plays a central role in the process of autophagy, and is critical to the formation of autophagosomes (Xie, Kang et al., 2015), while ATG5-mediated autophagy contributes to ferroptosis (Dixon, Lemberg et al., 2012; Hou, Xie et al., 2016). Unrestricted lipid peroxidation during ferroptosis could lead to plasma membrane rupture, and in cancer, ferroptosis also occurs in an autophagy-dependent manner (Liu, Kuang et al., 2020). Knocking down autophagy-related proteins including ATG3, ATG5, ATG7 and ATG13 inhibits ferroptosis (Gao, Monian et al., 2016; Hou, Xie et al., 2016; Chen, Kang et al., 2021). ATG5 is necessary for the fusion of autophagosomes in both canonical and non-canonical autophagy (Ye, Zhou et al., 2018; Fraiberg and Elazar 2020), and immune-related diseases happened when ATG5 gene mutated (Martin, Gupta et al., 2012; Zhang, Cheng et al., 2015). Here we demonstrated the value of ATG5 with multiple approaches (Figures 4B–G). Furthermore, the abundance of CD8^+^ T cells decreased in ATG5 high expression HNSCC patients, nevertheless the abundance of Tregs increased (*p* < 0.05, Figure 5A). High abundances of Tregs have been reported to be associated with adverse clinical outcomes (Sato, Olson et al., 2005; Beyer and Schultze 2006). Tregs create an immunosuppressive environment that impairs the activity of cytotoxic CD8^+^ T cells (Serrels, Lund et al., 2015). We further assessed the effect of ATG5 in HNSCC through predictive effects of ICB therapy. ICB therapy helps immune system recognize and attack tumor cells to achieve cancer treatment (Mahoney, Rennert et al., 2015), but in most cancer types only one-third patients benefit from ICB therapy (Sharma, Hu-Lieskovan et al., 2017). Using TIDE, which is a bioinformatic method (Jiang, Gu et al., 2018), it was predicted that ATG5 high expression group had a higher TIDE score than ATG5 low expression group in HNSCC. It means dysfunction of tumor-infiltrating cytotoxic T lymphocytes (CTL) and rejection of CTL by immunosuppressors are stronger in ATG5 high expression HNSCC, and the ICB efficacy is worse. These results suggest that ATG5 is an effective prognostic biomarker and a predictive target for immunotherapy efficacy in HNSCC and NPC.

G2M checkpoint is an important regulatory mechanism in eukaryotic cells that regulates the normal operation of the cell cycle, ensuring the integrity of the number of chromosomes, and most tumor cells have a G1 checkpoint, thus, controlling the proliferation of tumor cells to the G2M stage is a feasible tumor treatment regimen (Chen T et al., 2012). Controlling tumor cells at the G2M phase through the G2M checkpoints not only inhibits the proliferation and growth of tumors, but also induces the apoptosis of tumor cells by DNA damage (Chen, Landen et al., 2013). Previous studies showed some autophagy genes contribute to cell cycle (Qu, Yu et al., 2003; Lee, Kawai et al., 2012; Maskey, Yousefi et al., 2013), and research found lack of ATG5, autophagy in proximal tubular epithelial cells significantly aggravated cell cycle arrest in the G2/M phase (Li, Peng et al., 2016). We found that ATG5 expression was significantly positively correlated with the G2M checkpoint signaling pathway in HNSCC (Spearman coefficient = 0.41). We used GDSC, the largest publicly available pharmacogenomics database, to predict the sensitivity of G2M checkpoint inhibitor drugs in high and low ATG5 expression HNSCC samples, which based on sample transcriptomes. The results showed ATG5 high expression samples were more sensitive to G2M checkpoint inhibitors. An unexpected finding that G2M checkpoint inhibitors may have a therapeutic effect on ATG5 high expression HNSCC and NPC, but more basic studies and clinical trials are still needed to validate the therapeutic effect.

In conclusion, in order to explore the role of ferroptosis in immunotherapy in HNSCC and NPC, this study analyzed the expression of ferroptosis-related genes in HNSCC and NPC. We found that ferroptosis-related genes were positively correlated with PD-L1 and PD-L2 expression, and the types of immune infiltrates were clearly different between two groups of HNSCC patients with different ferroptosis-related genes expression level. Ferroptosis-related gene ATG5 played a key role in HNSCC and NPC treatment, as ATG5 high expression HNSCC patients showed poor OS, and had poor response and survival after ICB therapy. Furthermore, ATG5 expression was significantly positively correlated with G2M checkpoints in HNSCC (*p* < 0.001, Spearman coefficient = 0.41) and ATG5 high expression HNSCC patients had lower IC_50_ of G2M checkpoint inhibitors by predicted. Therefore, ATG5 has potential as a significant independent prognostic marker in HNSCC and NPC, and ATG5 is an important target in HNSCC and NPC treatment of ferroptosis, immunotherapy and G2M checkpoint inhibitor.

## Data Availability

Publicly available datasets were analyzed in this study. This data can be found here: TCGA dataset (https://portal.gdc.com) GEO database (https://www.ncbi.nlm.nih.gov/geo/).
