# Supplementary material for: Ferroptosis-related gene ATG5 is a novel prognostic biomarker in nasopharyngeal carcinoma and head and neck squamous cell carcinoma
Source: Front Bioeng Biotechnol. 2022 Sep 15;10:1006535. doi: 10.3389/fbioe.2022.1006535 (PMC9520473; doi:10.3389/fbioe.2022.1006535)
Supplement: Supplementary file 1 [file DataSheet4.PDF]

| | Chara | Number |
|---|---|---|
| **Status** | Alive | 286 |
| | Dead | 218 |
| **Age** | Mean (SD) | 61(11.9) |
| | Median [MIN, MAX] | 61[19,90] |
| **Gender** | Female | 134 |
| | Male | 370 |
| **Race** | American Indian | 2 |
| | Asian | 10 |
| | Black | 47 |
| | White | 430 |
| **pT stage** | T1 | 34 |
| | T2 | 146 |
| | T3 | 133 |
| | T4 | 25 |
| | T4a | 152 |
| | T4b | 3 |
| | TX | 11 |
| **pN stage** | N0 | 242 |
| | N1 | 81 |
| | N2 | 19 |
| | N2a | 18 |
| | N2b | 77 |
| | N2c | 41 |
| | N3 | 7 |
| | NX | 19 |
| **pM stage** | M0 | 479 |
| | M1 | 5 |
| | MX | 20 |
| **pTNM stage** | I | 25 |
| | II | 81 |
| | III | 91 |
| | IVA | 291 |
| | IVB | 13 |
| | IVC | 3 |
| **Grade** | G1 | 62 |
| | G2 | 301 |
| | G3 | 119 |
| | G4 | 2 |
| | GX | 17 |
| **Smoking** | Non-smoking | 113 |
| | Smoking | 381 |
