# Supplementary figures and images for: Ferroptosis-related gene ATG5 is a novel prognostic biomarker in nasopharyngeal carcinoma and head and neck squamous cell carcinoma
Source: Front Bioeng Biotechnol. 2022 Sep 15;10:1006535. doi: 10.3389/fbioe.2022.1006535 (PMC9520473; doi:10.3389/fbioe.2022.1006535)

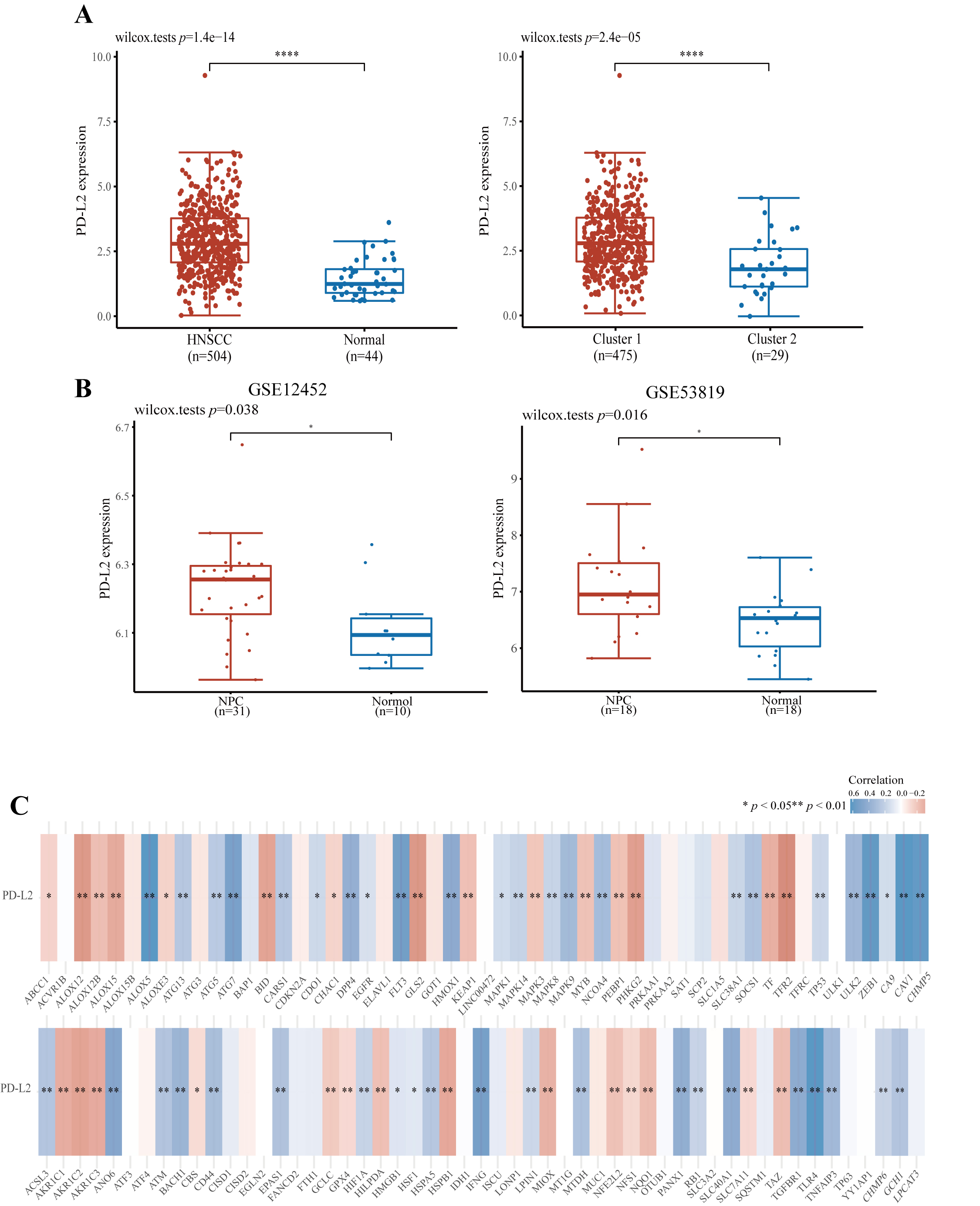

Supplement: Supplementary file 2 [file Image1.JPEG]
