# Supplementary material for: Ferroptosis-related gene ATG5 is a novel prognostic biomarker in nasopharyngeal carcinoma and head and neck squamous cell carcinoma
Source: Front Bioeng Biotechnol. 2022 Sep 15;10:1006535. doi: 10.3389/fbioe.2022.1006535 (PMC9520473; doi:10.3389/fbioe.2022.1006535)
Supplement: Supplementary file 4 [file DataSheet3.PDF]

	P values			P values			P values	
**CA9**	2.12E-24	up	**FTMT**	3.87E-08	up	**MT1G**	0.129643	up
**SOCS1**	9.33E-22	up	**CBS**	6.70E-08	up	**ZEB1**	0.129894	up
**HSF1**	1.03E-21	up	**MAPK8**	9.20E-08	up	**ALOX12B**	0.151401	up
**CISD2**	2.85E-20	up	**ANO6**	1.17E-07	up	**AKR1C2**	0.198728	up
**SLC3A2**	7.15E-20	up	**TFR2**	1.81E-07	up	**ISCU**	0.205044	up
**BID**	1.07E-17	up	**KEAP1**	3.68E-07	up	**ATF3**	0.219597	up
**PHKG2**	1.77E-17	up	**MAPK1**	7.47E-07	up	**NFE2L2**	0.272404	up
**TF**	7.52E-17	down	**GOT1**	8.03E-07	down	**TP53**	0.275452	up
**ULK1**	1.04E-16	up	**BAP1**	2.17E-06	up	**HSPB1**	0.29882	up
**HSPA5**	5.78E-16	up	**ALOX12**	2.95E-06	down	**LINC00472**	0.413034	up
**TGFBR1**	1.03E-15	up	**MAPK3**	3.12E-06	down	**ACSL3**	0.455612	up
**ATG13**	1.33E-15	up	**MAPK14**	4.30E-06	up	**FLT3**	0.479335	up
**MTDH**	4.41E-15	up	**RB1**	1.04E-05	up	**IDH1**	0.539183	up
**ELAVL1**	9.10E-15	up	**HILPDA**	1.09E-05	up	**HMOX1**	0.542469	up
**TAZ**	1.56E-14	up	**MAPK9**	1.11E-05	up	**ALOX5**	0.549731	up
**TFRC**	2.20E-14	up	**BACH1**	2.07E-05	up	**ATG7**	0.58129	up
**TP63**	2.82E-14	up	**CDKN2A**	2.90E-05	up	**GLS2**	0.62394	up
**NFS1**	3.74E-14	up	**SLC7A11**	3.04E-05	up	**CHMP6**	0.65254	up
**HIF1A**	5.56E-14	up	**GCLC**	6.01E-05	up	**TLR4**	0.764688	up
**CAV1**	7.64E-14	up	**PRKAA1**	9.81E-05	up	**AKR1C1**	0.985346	up
**ATG3**	8.89E-14	up	**SCP2**	0.000106	down	**DPP4**	0.07353	up
**LONP1**	1.10E-13	up	**SAT1**	0.000156	up			
**CARS1**	1.11E-13	up	**ATF4**	0.000187	up			
**SLC1A5**	1.28E-13	up	**ALOXE3**	0.000209	up			
**OTUB1**	3.35E-13	up	**ALOX15**	0.00063	down			
**ATG5**	3.74E-13	up	**GPX4**	0.000768	up			
**FANCD2**	1.03E-12	up	**SQSTM1**	0.001624	up			
**PANX1**	2.95E-12	up	**CHAC1**	0.00259	down			
**CD44**	5.08E-12	up	**YY1AP1**	0.003387	up			
**MIOX**	1.18E-11	up	**ATM**	0.00432	up			
**LPIN1**	4.68E-11	down	**MUC1**	0.01032	down			
**PRKAA2**	7.04E-11	down	**ULK2**	0.01035	up			
**EGLN2**	1.04E-10	up	**EPAS1**	0.012581	up			
**TNFAIP3**	1.30E-10	up	**MYB**	0.021123	down			
**ABCC1**	6.46E-10	up	**CISD1**	0.024	up			
**FTH1**	6.63E-10	up	**LPCAT3**	0.024563	up			
**CDO1**	1.83E-09	down	**ACVR1B**	0.025561	up			
**PEBP1**	2.74E-09	down	**ALOX15B**	0.030479	down			
**IFNG**	5.83E-09	up	**GCH1**	0.042631	up			
**CHMP5**	8.04E-09	up	**NCOA4**	0.043916	down			
**HMGB1**	1.23E-08	up	**NQO1**	0.047576	up			
**SLC38A1**	1.36E-08	up	**AKR1C3**	0.05773	up			
**EGFR**	2.65E-08	up	**SLC40A1**	0.066771	up			
